# Bone marrow stromal cells enhance differentiation of acute myeloid leukemia induced by pyrimidine synthesis inhibitors

**DOI:** 10.1152/ajpcell.00413.2024

**Published:** 2024-09-16

**Authors:** Tomislav Smoljo, Hrvoje Lalic, Vilma Dembitz, Barbara Tomic, Josip Batinic, Radovan Vrhovac, Antonio Bedalov, Dora Visnjic

**Affiliations:** ^1^Laboratory for Cell Biology, Department of Physiology, Croatian Institute for Brain Research, University of Zagreb School of Medicine, Zagreb, Croatia; ^2^Department of Laboratory Immunology, Clinical Department of Laboratory Diagnostics, University Hospital Center Zagreb, Zagreb, Croatia; ^3^Division of Hematology, Department of Internal Medicine, University Hospital Centre Zagreb, Zagreb, Croatia; ^4^Department of Internal Medicine, University of Zagreb School of Medicine, Zagreb, Croatia; ^5^Clinical Research Division, Fred Hutchinson Cancer Research Centre, Seattle, Washington, United States

**Keywords:** acute myeloid leukemia, AICAr, bone marrow stromal cells, brequinar, differentiation

## Abstract

Acute myeloid leukemia (AML) is a heterogeneous group of hematological malignancies characterized by differentiation arrest, high relapse rates, and poor survival. The bone marrow (BM) microenvironment is recognized as a critical mediator of drug resistance and a primary site responsible for AML relapse. Our previous study reported that 5-aminoimidazole-4-carboxamide ribonucleoside (AICAr) induces AML cell differentiation by inhibiting pyrimidine synthesis and activating Checkpoint kinase 1. Although the protective effect of BM stroma on leukemia cells in response to cytotoxic drugs is well-documented, its effect on AML differentiation remains less explored. In this study, we investigated the impact of stromal cell lines and primary mesenchymal stromal cells (MSCs) on AML cell line differentiation triggered by AICAr and brequinar, a known dihydroorotate dehydrogenase (DHODH) inhibitor. Our findings indicate that the mouse MS-5 stromal cell line, known for its cytoprotective effects, does not inhibit AML cell differentiation induced by pyrimidine synthesis inhibitors. Interestingly, AICAr caused morphological changes and growth arrest in MS-5 stromal cells via an AMP-activated protein kinase (AMPK)-dependent pathway. Human stromal cell lines HS-5 and HS-27, as well as primary MSCs isolated from patient bone marrow, were superior in promoting AML differentiation compared with mouse cells in response to AICAr and brequinar, with the inhibitors not significantly affecting the stromal cells themselves. In conclusion, our study highlights the supportive role of human BM MSCs in enhancing the differentiation effects of pyrimidine synthesis inhibitors on AML cells, suggesting that AML treatment strategies focusing on differentiation rather than cell killing may be successful in clinical settings.

**NEW & NOTEWORTHY** This study is the first to demonstrate that human stromal cell lines and primary mesenchymal stromal cells from patients enhance the in vitro differentiation of acute myeloid leukemia (AML) cells induced by pyrimidine synthesis inhibitors, 5-aminoimidazole-4-carboxamide ribonucleoside (AICAr), and brequinar. Furthermore, this is the first report to show that AICAr affects mouse bone marrow stromal cells by activating AMP-activated protein kinase (AMPK) and that human stromal cells are superior to mouse cells for testing the effects of drugs on AML differentiation.

## Graphical abstract

**Figure:**
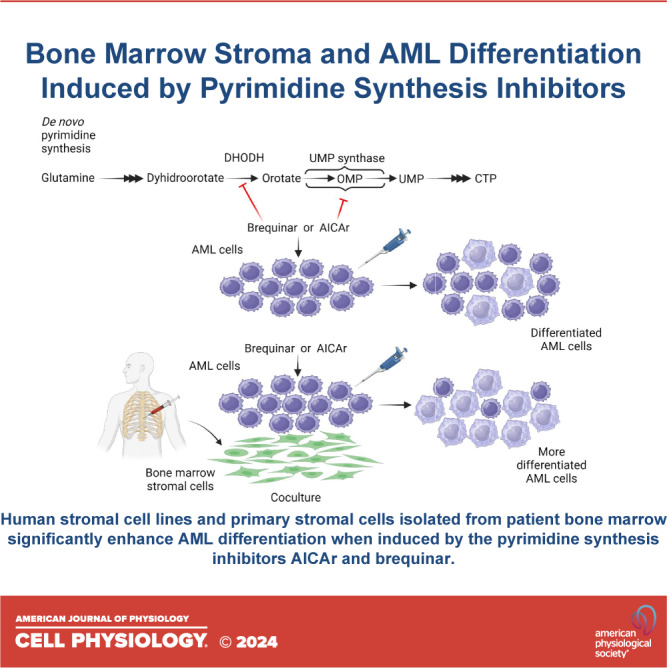

## INTRODUCTION

AMP-activated protein kinase (AMPK) is a highly conserved serine/threonine kinase that serves as a key regulator of cellular metabolism, responding to increases in the AMP/ATP ratio (1). 5-Aminoimidazole-4-carboxamide ribonucleoside (AICAr or acadesine) is extensively used as a pharmacological agonist to stimulate AMPK activity. Exogenously administered AICAr enters cells via adenosine transporters and is subsequently phosphorylated by adenosine kinase into 5-aminoimidazole-4-carboxamide ribonucleotide (AICAR), also referred to as ZMP, which acts as an AMP mimetic (2, 3). Endogenous AICAR is an intracellular intermediate in de novo purine biosynthesis, known to accumulate in Lesch–Nyhan syndrome and other purine synthesis disorders (4). Although AICAr is commonly used as an AMPK activator in studies concerning metabolism and the insulin signaling pathway, an increasing number of studies have demonstrated that many effects attributed to AICAr are actually AMPK-independent (5).

Acute myeloid leukemia (AML) is an aggressive hematological malignancy marked by the clonal expansion of myeloid blasts arrested in differentiation, making differentiation-inducing therapies a promising treatment strategy (6). AMPK modulators have demonstrated cytotoxic activity in hematological malignancies, yet their role in the differentiation of AML remains relatively unexplored (7–9). Our recent studies, aimed at investigating metabolic changes during AML differentiation, have revealed that AICAr alone is capable of inducing differentiation in various AML cell lines (10, 11), as well as in a subset of primary blasts isolated from the bone marrow of patients with AML (12). The effects of AICAr on monocytic cell lines were found not to be AMPK-dependent, as both growth arrest and differentiation were preserved in AML cells with siRNA-downregulated AMPK (5, 10). AICAr-induced differentiation was inhibited by the downregulation of checkpoint kinase 1 (Chk1), and activation of Chk1 was induced by defects in pyrimidine synthesis (11). AICAr inhibits pyrimidine synthesis at the level of UMP synthase, which acts downstream of dihydroorotate dehydrogenase (DHODH), thereby sharing a similar mechanism of leukemia differentiation as that observed with brequinar, a well-known DHODH inhibitor (13). To further investigate the role of the DNA damage response in the differentiation of leukemia cells, our recent study examined the differentiation mechanism in response to low-dose cytarabine (LDAC). The proposed mechanism of cytarabine (AraC) action involves its rapid conversion into cytosine arabinoside triphosphate (AraCTP), which disrupts DNA replication through multiple mechanisms, primarily by incorporating into the DNA molecule (14). Unlike AICAr and brequinar, cytarabine-mediated differentiation was not abolished by the addition of nucleosides. However, the effects on the expression of differentiation markers were decreased by the pharmacological and genetic downregulation of Chk1 (15). In addition, cytarabine induced differentiation ex vivo in a subset of primary AML samples that are sensitive to AICAr and DHODH inhibitor (12, 15), suggesting that differentiation in response to LDAC may share mechanisms with those used by de novo pyrimidine synthesis inhibitors.

The bone marrow (BM) microenvironment plays a crucial role in normal hematopoiesis and may significantly contribute to drug resistance in vivo. Large-scale compound screens are typically conducted in monocultures of AML cells, but these should be followed by evaluations of potential stroma-mediated resistance in coculture systems (16, 17). BM stroma is known to confer protective effects on leukemia cells in response to various cytotoxic drugs, including cytarabine (18, 19). However, the effect of stroma on AML differentiation is less studied, with drug responses reported to either decrease or increase differentiation, depending on the agent used. The coculture of AML and stromal cells reduced all-*trans* retinoic acid (ATRA)-mediated differentiation in both AML cell lines and primary cells (20). In contrast, the coculture of primary blasts with human BM stroma showed that FLT3 inhibition with quizartinib induced cell-cycle arrest and differentiation rather than apoptosis (21). Our recent study, which investigated the effects of the murine BM stromal cell line on AML differentiation, confirmed the protective effect of stromal MS-5 cells against apoptosis induced by high doses of AraC. In addition, it revealed that the presence of MS-5 cells decreased the differentiation of both AML cell lines and primary blasts induced by low doses of AraC (22).

The reason why MS-5 cells are still used in coculture models with human AML despite being of murine origin is because they are a user-friendly system that exhibits contact inhibition. These cells were initially used in coculture with leukemic cells HL-60 to unveil the stromal protective effect on the cytotoxicity of cytarabine (18). However, it is expected that the effects of mouse and human stromal cells may differ, particularly as these effects are often attributed to cytokines, some of which are species-specific (23). HS-5 and HS-27 are two functionally distinct human BM stromal cell lines immortalized by transduction with the human papillomavirus E6/E7. They differ in their cytokine secretion profiles and their ability to support myeloid colonies (24, 25). However, the immunophenotype of HS-5 and HS-27a cell lines resembles that of bone marrow-derived mesenchymal stromal cells (BM-MSCs) from both patients with AML and healthy donors (26).

In this study, we examined the impact of established stromal cell lines as well as primary mesenchymal stromal cells (MSCs) on the differentiation of AML cell lines in response to AICAr and brequinar. Our results demonstrate that both human bone marrow stromal cell lines and primary MSCs, when cocultured with AML cells, enhance AML cell differentiation induced by de novo pyrimidine synthesis inhibitors.

## MATERIALS AND METHODS

### Reagents

The reagents used are detailed in Supplemental Table S1.

### Cell Lines and Cocultures

Human AML cell lines U937 and THP-1 were maintained in RPMI 1640 supplemented with 10% fetal bovine serum, 2 mM l-glutamine, 50 U/mL penicillin, and 50 μg/mL streptomycin. The mouse stromal MS-5 cell line was cultured in alpha-MEM (with ribo- and deoxyribonucleosides), supplemented with 10% fetal bovine serum, 2 mM l-glutamine, 2 mM sodium pyruvate, 50 U/mL penicillin, and 50 μg/mL streptomycin. Human stromal cell lines HS-5 and HS-27 were maintained in DMEM supplemented with 10% fetal bovine serum, 2 mM l-glutamine, 50 U/mL penicillin, and 50 μg/mL streptomycin. All cell lines were cultured at 37°C in a humidified atmosphere containing 5% CO_2_.

For the experiments, MS-5, HS-5, or HS-27 stromal cells were seeded in alpha MEM or DMEM at a starting concentration of 20 × 10^3^/cm^2^ in 6-well plates or 25 cm^2^ flasks 24 h before the addition of AML cells. After 24 h of incubation and establishment of the stromal monolayer, the medium was replaced with supplemented RPMI 1640. Exponentially growing AML cell lines were seeded at a starting concentration of 0.2 × 10^6^/mL in 6-well plates or 0.3 × 10^6^/mL in 25 cm^2^ flasks. The agents were added as described in the figure legends.

### Primary Mesenchymal Stromal Cells and Cocultures

The study was performed according to the Declaration of Helsinki and approved by the Institutional Review Board of the University of Zagreb School of Medicine (Approval Number: 641-01/23-02/01) and University Hospital Center Zagreb (Approval Number: 02/013 JG). Bone marrow samples were obtained upon written informed consent from patients suspected of having AML, before the initiation of any treatment. The samples were evaluated for *FLT3* mutations and cytogenetic abnormalities as part of routine diagnostic procedures. Subsequently, samples exhibiting normal karyotypes underwent additional testing for *NPM1* mutations. Further analyses, including assessments for *BCR-ABL*, *RUNX1/RUNX1T1*, *PML-RARA*, *CBFB/MYH11*, and *KMT2AAFF1/MLLT3* mutations, were conducted based on the French-American-British (FAB) subtype classification. Healthy human stroma was obtained from a bone marrow donor in accordance with the standard administrative and medical protocols established by the Clinical Hospital Center Zagreb for bone marrow donation.

Diagnostic bone marrow aspirates were separated using NycoPrep 1.077, as previously described (15, 22, 27). Isolation and culture of MSCs from patients were performed as previously described (28). In brief, after an overnight incubation, the nonadherent cells were removed, and the adherent cells were maintained in DMEM medium supplemented with 10% FBS, 2 mM glutamine, 50 U/mL penicillin, and 50 µg/mL streptomycin. Complete media changes were performed every 3–4 days after visually confirming the presence of attached cells. When the attached cells reached ∼95% confluence, they were passaged. For passaging, the cells were washed once with 1× PBS, detached using 0.25% (wt/vol) trypsin and 0.02% (wt/vol) EDTA, and then passaged at a 1:2 ratio. MSCs obtained from the bone marrow sample Pt03 were cryopreserved in liquid nitrogen, whereas MSCs from the bone marrow samples Pt38 and Pt40 were used without cryopreservation.

MSCs were used at passages 3 or 4 in the subsequent experiments. Purified MSCs were verified to express the characteristic markers CD73 and CD105, while lacking the presence of CD45 (29). MSCs were seeded in 12-well plates at a concentration of 20 × 10^3^/cm^2^. After 24 h, media was replaced with supplemented RPMI 1640, and the cells were treated either alone or in the presence of U937 cells, initially seeded at a concentration of 0.2 × 10^6^/mL. The agents were added as described in the figure legends, with each condition assessed in triplicate.

The characteristics of the patients are detailed in Supplemental Table S2.

### Cell Proliferation and Apoptosis

The number of viable cells was determined using a hemocytometer and the trypan blue exclusion method at the specified time points. Apoptosis was assessed using an annexin V—fluorescein isothiocyanate (FITC) kit following the manufacturer’s instructions. Samples were then analyzed for the percentage of annexin V-FITC and propidium iodide (PI)-positive cells using the FACSCanto II flow cytometer (BD Biosciences, San Jose, CA) and FlowJo v.10 platform. In brief, the double-negative region was identified on the Annexin V and PI fluorescence plot for the entire dataset. Debris was gated on a plot of forward scatter (FSC) versus side scatter (SSC) for the double-negative cells. This debris gate was then inverted (i.e., excluding debris) and applied to the annexin V-FITC versus PI plot. Quadrants were drawn to define the four populations present (Supplemental Fig. S1).

### Immunophenotyping

At the end of incubation, U937 and THP-1 cells were collected, washed, aliquoted into three tubes, and incubated with Fc Receptor Blocking solution for 10 min. Cells were stained with either anti-CD11b-FITC, anti-CD64-FITC, or their respective isotypic control for additional 20 min at room temperature in the dark.

For the analysis of MS-5, HS-5, and HS-27 stromal cell lines, as well as primary MSCs, nonadherent cells and media were removed. Adherent cells were then harvested through trypsinization, washed in RPMI 1640, and resuspended in PBS containing 1% BSA before being aliquoted into three tubes. Subsequently, the cells were incubated with an Fc Receptor Blocking solution on ice for 10 min. Following this, MS-5 cells in the first tube were stained with anti-CD45-FITC, anti-CD11b-PE, anti-CD90.2-PE-Cy7, and anti-CD34-eFluor 660 antibodies. Cells in the second tube were stained with anti-Ly-6A/E (Sca-1)-FITC, anti-CD44-PE, anti-CD105-PE-Cy7, and anti-CD140a(PDGFRα)-APC antibodies. HS-5, HS-27, and primary MSCs in the first tube were stained with anti-CD73-FITC, anti-CD45-APC, anti-CXCR4(CD184)-PE, and anti-CD105-PE-Cy7 antibodies. Human stromal cells in the second tube were stained with anti-CD140a-PE, anti-CD90-PE-Cy7, and anti-CD34-APC antibodies. Cells in the third tube were stained with their respective isotypic controls. Each tube was then incubated for additional 20 min at room temperature in the dark.

Nonviable cells were excluded based on FSC versus SSC gating and through 7-AAD staining. Doublet exclusion was conducted by plotting the height against the area for FSC. Flow cytometry analyses were carried out using a FACSCanto II flow cytometer (BD Biosciences, San Jose, CA), and the data obtained were analyzed using the FlowJo v.10 platform. The mean fluorescence intensity (MFI) of the sample was determined by subtracting the MFI levels of isotypic controls from those of the cells stained with CD-specific antibodies.

### Intracellular Reactive Oxygen Species

Intracellular reactive oxygen species (ROS) levels were assessed by staining cells with dihydrorhodamine 123 (DHR123). Following the incubation period, the cells were harvested in PBS and then treated with either 100 μM DHR123 or left untreated, followed by incubation at 37°C in the dark for 15 min. Subsequently, the cells were analyzed using flow cytometry with the FACSCanto II instrument (BD Biosciences, San Jose, CA), and MFI of the sample was determined using the FlowJo v.10 platform.

### Morphological Analysis

At the end of incubation, U937 and THP-1 cells were collected, washed, counted, and resuspended in PBS. Subsequently, 50,000 cells per sample were cytospun onto microscopic slides using the StatSpin Cytofuge 2 (Beckman Coulter, Marseille, France) at 1,000 rpm for 2 min and allowed to dry overnight.

For the analysis of adherent stromal cell lines and primary cells, cell culture media were removed, plates were washed with PBS, and then left to dry overnight.

Air-dried slides and plates were stained with May-Grünwald stain (50% working solution, 5 min) followed by Giemsa stain (10% working solution, 20 min). Morphological examination was conducted using an AxioVert 200 microscope, and images were captured with an AxioCam MRc 5 camera and ZEN software, blue edition (Carl Zeiss AG, Oberkochen, Germany). The images of leukemia cells stained with May-Grünwald-Giemsa were processed using Fiji (ImageJ) with the Trainable Weka Segmentation plugin (30, 31). Nuclei and cytosol were manually annotated, and the classifier was trained and applied to segment these regions. Binary masks were generated, and the areas of nuclei and cytosol were measured. The nuclear/cytoplasmic (N/C) ratio was calculated by dividing the total nuclei area by the total cytosol area.

### Total Cell Lysates and Western Blot

For the experiments, exponentially growing U937 cells were collected, plated at the concentration of 0.3 × 10^6^/mL in 25 cm^2^ flasks, and incubated as described in the figure legends. At the end of the incubation period, cells were harvested and suspended in cell lysis buffer supplemented with freshly added 1 mM phenylmethylsulfonyl fluoride and 1 μM microcystin, then incubated on ice for 10 min. Following this, cells were disrupted by passing through a 23-gauge needle seven times and further incubated on ice for an additional 10 min. Subsequently, the cell lysates were centrifuged at 14,000 *g* at 4°C for 10 min, and the resulting supernatants were collected and stored at −80°C. The protein concentration of each sample was determined colorimetrically using Bradford reagent and measured at 595 nm using Eppendorf Biophotometer Plus.

Equal amounts of protein extracts (50 μg/well) were combined with LDS Sample Buffer (4X) and subjected to electrophoresis on a 4–12% SDS-polyacrylamide gel. Subsequently, the separated proteins were transferred to a PVDF membrane using the Mini-PROTEAN Tetra electrophoresis system (Bio-Rad Laboratories, Hercules, CA). After transfer, the membranes were incubated in Tris-buffered saline-Tween 20 (TBST) and 5% wt/vol nonfat dry milk (a blocking buffer) for 30 min at room temperature. They were then washed three times for 5 min each with TBST. Primary antibodies, including anti-Chk1, anti-pChk1 (S345), anti-AMPK, anti-phospho-AMPK-alpha (T172) were used at a dilution of 1:1,000, and monoclonal anti-β-actin was used at a dilution of 1:40,000. Membranes and primary antibodies were incubated in 10 mL of primary antibody dilution buffer (TBST with 5% BSA) with gentle agitation overnight at 4°C. Following the primary antibody incubation, membranes were washed three times for 5 min each with 20 mL of TBST. Membranes were then incubated with the appropriate anti-mouse IgG (dilution 1:2,000) or anti-rabbit IgG (dilution 1:2,000) horseradish peroxidase (HRP)-linked secondary antibodies in 10 mL of secondary antibody dilution buffer (TBST with 5% wt/vol nonfat dry milk) for 120 min at room temperature. Protein bands were visualized using the SuperSignal West Pico PLUS chemiluminescent substrate and the ChemiDoc MP Imaging System (Bio-Rad Laboratories, Hercules, CA), with analysis performed by Image Lab software (Bio-Rad Laboratories, Hercules, CA).

### Statistical Analysis

The data are presented as means ± SE. Student’s *t* test was used for monoculture experiments, whereas one-way analysis of variance (ANOVA), followed by Tukey’s multiple comparison test, was performed for coculture experiments using GraphPad Prism v. 6.07. The results were considered statistically significant if *P* was <0.05. Experiments were not performed in a blinded manner.

## RESULTS

### Murine Stromal Cells MS-5 Do Not Inhibit Differentiation Induced by AICAr and Brequinar in Two Monocytic Cell Lines

**Figure 1.**
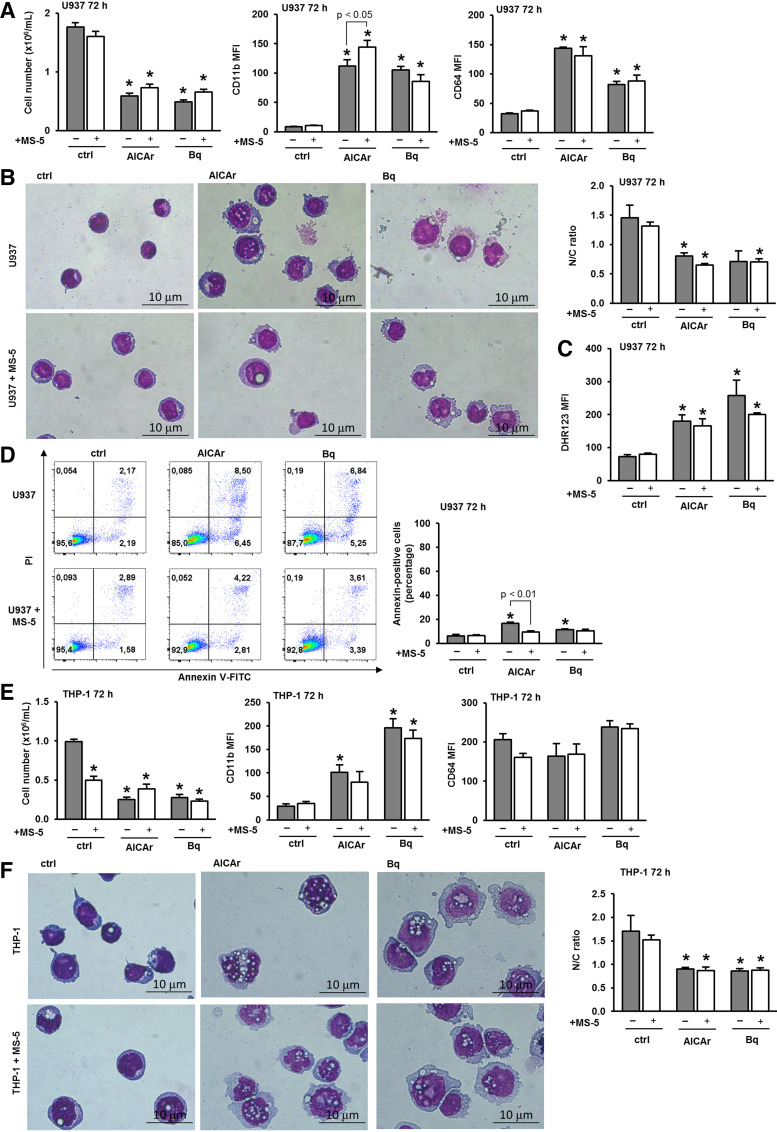
The murine stromal cell line MS-5 does not inhibit the induction of differentiation in U937 and THP-1 cells in response to AICAr and brequinar. U937 cells were cultured either alone or in the presence of the MS-5 stromal cell line and were left untreated (ctrl) or treated with AICAr (0.2 mM) or brequinar (Bq) (0.5 μM) for 72 h. *A*: the number of viable U937 cells and the expression of differentiation markers. The mean fluorescence intensity (MFI) of CD11b and CD64 was calculated as described in materials and methods. *B*: representative May-Grünwald-Giemsa stained cytospin preparations of U937 cells (×100 magnification) after 72 h of incubation. The nuclear/cytoplasmic (N/C) ratio was calculated as described in materials and methods. *C*: DHR123 fluorescence in U937 cells analyzed by flow cytometry as described in materials and methods. *D*: Representative dot plots of cells stained with annexin V-FITC/propidium iodide (PI) and the percentage of annexin-positive U937 cells analyzed by flow cytometry. *E*: THP-1 cells were cultured either alone or in the presence of the MS-5 stromal cell line and were left untreated (ctrl) or treated with AICAr (0.2 mM) or brequinar (Bq) (0.5 μM) for 72 h. The number of viable cells and the expression of differentiation markers. The mean fluorescence intensity (MFI) of CD11b and CD64 was calculated as described in materials and methods. *F*: representative May-Grünwald-Giemsa stained cytospin preparations of THP-1 cells (×100 magnification) after 72 h of incubation. The nuclear/cytoplasmic (N/C) ratio was calculated as described in MATERIALS AND METHODS. Results represent the means ± SE (error bars) of at least three independent experiments. *P < 0.05 (ANOVA and Tukey) compared with control (ctrl). AICAr, 5-aminoimidazole-4-carboxamide ribonucleoside; DHR123, dihydrorhodamine 123; FITC, fluorescein isothiocyanate.

To examine the effects of BM stromal cells on AML differentiation induced by pyrimidine synthesis inhibitors, we initially used the murine adherent fibroblastic MS-5 stromal cell line. This cell line had previously been used to examine the effects of stromal cells on AML cells treated with varying doses of cytarabine (18, 22). MS-5 cells were plated in 6-well plates and incubated either alone or in combination with monocytic AML cell line U937, which were added after 24 h. AICAr and the DHODH inhibitor brequinar were added at doses previously demonstrated to induce differentiation (11, 12). After 72 h, both AICAr and brequinar decreased the number of viable cells and induced the expression of differentiation markers. The presence of MS-5 cells did not prevent the differentiation of U937 cells induced by AICAr and brequinar; rather, it even increased the expression of CD11b in the cells treated with AICAr (Fig. 1*A*). As illustrated in Fig. 1*B*, AICAr and brequinar induced a decrease in the nuclear/cytoplasmic ratio, and these effects were still evident in the presence of MS-5 cells. In addition, the presence of MS-5 cells had no effect on the increase in reactive oxygen species (Fig. 1*C*). As shown in Fig. 1*D*, both AICAr and brequinar induced a small but significant increase in the percentage of annexin-positive cells, and these effects were inhibited by the presence of stromal cells. Therefore, we concluded that the presence of MS-5 cells increased the number of viable cells by decreasing apoptosis, but did not prevent an increase in the expression of differentiation markers induced by pyrimidine synthesis inhibitors in U937 cells.

We further investigated whether similar effects of the presence of mouse stromal cells could be observed in a coculture with another human leukemia monocytic cell line. THP-1 cells were incubated either alone or in a coculture with MS-5 cells and then treated with the same doses of pyrimidine synthesis inhibitors. As shown in Fig. 1*E*, both AICAr and brequinar reduced the number of viable THP-1 cells and increased the expression of CD11b, while having no effect on the expression of CD64, as previously described (11). This lack of effect on CD64 is likely because THP-1 cells are SAM domain and HD domain-containing protein 1 (SAMHD1)-proficient AML cells, which are described as being better able to counteract changes in the dNTP pool induced by the drug treatment (32). The presence of MS-5 cells had no statistically significant effects on the viability and the expression of CD11b (Fig. 1*E*). Furthermore, the presence of MS-5 cells did not abolish the morphological changes and the decrease in the nuclear/cytoplasmic ratio of THP-1 cells induced by AICAr and brequinar (Fig. 1*F*). Therefore, we confirmed the conclusion obtained in U937 cells that the presence of the mouse stromal cell line cannot prevent the differentiation of monocytic cell lines induced by the inhibitors of pyrimidine synthesis.

### AICAr Inhibits Proliferation and Induces Fibrocyte-like Changes in MS-5 Stromal Cells, and These Effects Cannot Be Abolished by the Addition of Nucleosides

**Figure 2.**
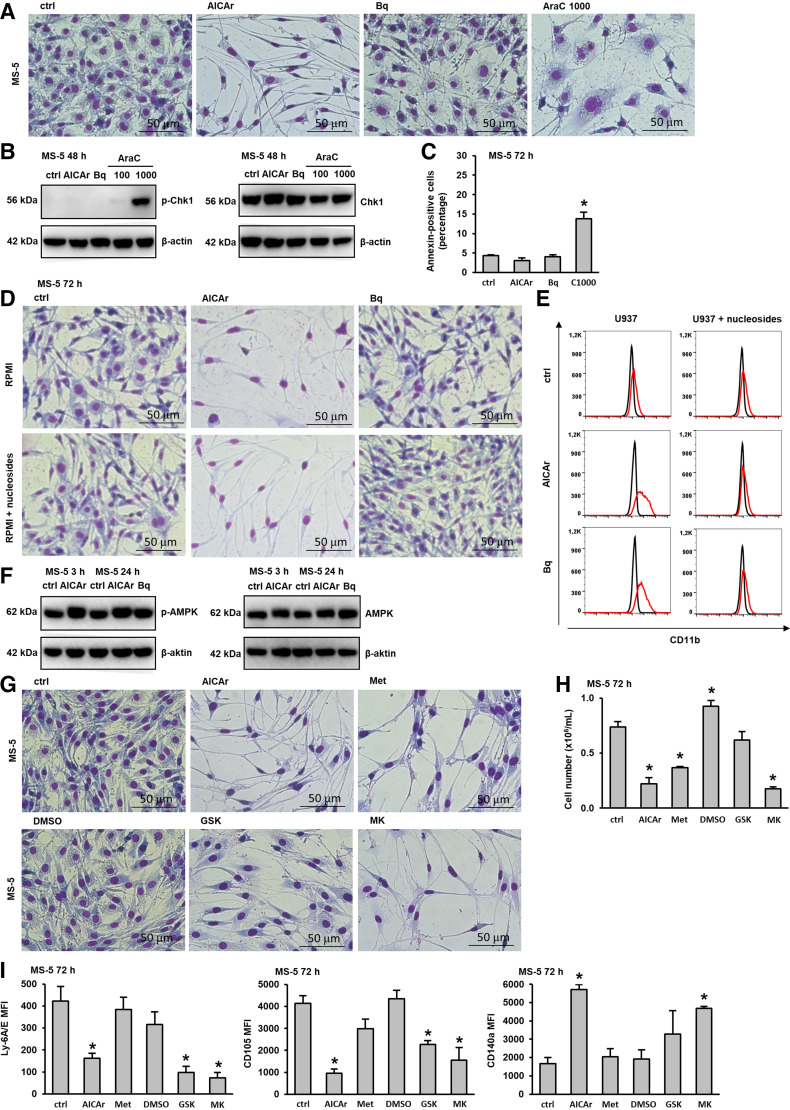
AICAr induces phenotypic changes in MS-5 cells that cannot be abolished by the addition of nucleosides but are mimicked by AMPK agonists. MS-5 stromal cells were seeded in 6-well plates and allowed to adhere for 24 h. After 24 h, the media were replaced, and the cells were incubated untreated (ctrl) or treated with AICAr (0.2 mM), brequinar (Bq) (0.5 μM), and AraC (100 or 1000 nM). *A*: representative images of May-Grünwald-Giemsa stained MS-5 cells (×40 magnification) 72 h after the addition of the tested agents. *B*: total cell lysates from MS-5 cells were isolated after 48 h and analyzed by Western blotting to determine the levels of Ser345-phosphorylated Chk1 and total Chk1. Representative immunoblots from three independent experiments are shown. *C*: the percentage of annexin-positive MS-5 cells 72 h after the addition of the tested agents. *D*: representative images of May-Grünwald-Giemsa stained MS-5 cells (×40 magnification) incubated for 72 h with the tested agents, with or without the addition of nucleosides. *E*: the expression of CD11b in U937 cells incubated for 72 h with the tested agents, with or without the addition of nucleosides. Representative histograms are shown, with a red line representing the expression of CD11b and a black line representing the isotypic control. *F*: total cell lysates from MS-5 cells were isolated after 3 and 24 h and analyzed by Western blotting to determine the levels of Thr172-phosphorylated AMPK and total AMPK. Representative immunoblots from three independent experiments are shown. MS-5 stromal cells incubated with media alone (ctrl), AICAr (0.2 mM), brequinar (Bq) (0.5 μM), metformin (Met) (15 mM), DMSO (0,1%), GSK-621 (GSK) (30 μM) or MK-8722 (MK) (10 μM). *G*: Representative May-Grünwald-Giemsa stained MS-5 cells (×40 magnification) 72 h after the addition of the tested agents. *H*: the number of viable MS-5 cells and *I*: immunophenotype of MS-5 cells analyzed as described in MATERIALS AND METHODS. Results represent the means ± SE (error bars) of at least three independent experiments. *P < 0.05 (Student’s *t* test) compared with control (ctrl). AICAr, 5-aminoimidazole-4-carboxamide ribonucleoside; AMPK, AMP-activated protein kinase.

Several studies have revealed that drugs that are cytotoxic to leukemia cells affect the viability and function of the stromal cells themselves (33, 34). Our recent study revealed that cytarabine used at a cytotoxic dose (1,000 nM) affects the viability and function of MS-5 stromal cells (22). To assess the potential effects of AICAr and brequinar on MS-5 stromal cells, the stromal cells were incubated in the presence of pyrimidine synthesis inhibitors for 72 h and compared with the effects of a cytotoxic dose of cytarabine. As shown in Fig. 2*A*, cytarabine reduced the proliferation of stromal cells and induced morphological changes, as previously described (22). No significant changes were induced by incubation with brequinar at a dose of 0.5 μM, which induces differentiation of AML cells. However, treatment with 0.2 mM AICAr inhibited growth and induced a fibrocyte-like appearance in MS-5 cells. In AML cells, AICAr, brequinar, and cytarabine induced the activation of Chk1, and the activation of the DNA damage signaling pathway participated in drug-induced differentiation (11, 15). To test for the possible activation of Chk1 in stromal cells, MS-5 cells were incubated with AICAr, brequinar, and two doses of cytarabine for 48 h. Total cell lysates were then analyzed by Western blot for the phosphorylation of Chk1 on Ser345. As shown in Fig. 2*B*, cytarabine in high dose induced the activation of Chk1, but no increase in phosphorylation was observed in lysates of MS-5 cells treated with AICAr and brequinar. Moreover, high-dose cytarabine (1,000 nM) induced an increase in the percentage of annexin-positive cells, as previously described (22), but no increase in apoptosis was observed in MS-5 cells treated with AICAr and brequinar (Fig. 2*C*).

The difference in the effects of AICAr and brequinar on the proliferation and morphology of MS-5 stromal cells raised the possibility that AICAr-mediated effects on stroma did not depend on the inhibition of de novo pyrimidine synthesis. Our previous study demonstrated that the effects of both AICAr and brequinar on the differentiation of AML cells can be completely abolished by the exogenous addition of a mixture of nucleosides (11). To test whether the addition of nucleosides could alter the response of MS-5 cells to AICAr similar to the response of AML cells, a mixture of nucleosides (A, G, C, T, and U) was added to either MS-5 or U937 cells before the addition of AICAr and brequinar. As shown in Fig. 2*D*, nucleosides had no effects on AICAr-induced changes in stroma, although their presence completely prevented differentiation of AML cells induced by AICAr and brequinar (Fig. 2*E*).

Therefore, we concluded that AICAr inhibits proliferation and induces fibrocyte-like changes in MS-5 stromal cells by a mechanism that differs from the inhibition of pyrimidine synthesis and activation of DNA damage signaling pathway responsible for differentiation of AML cells.

### AICAr Activated AMPK in MS-5 Cells, and the Phenotypic Changes Induced by AICAr Were Mimicked by AMPK Activators

AICAr is known to exert both AMPK-dependent and AMPK-independent effects in various cells (5). Our previous study showed that AICAr activates AMPK in U937 cells, but the effects on differentiation were neither abolished by AMPK downregulation (10) nor mimicked by the addition of an AMPK agonist (11). To test for the activation of AMPK in MS-5 cells, the levels of total and phosphorylated AMPK were determined in lysates of MS-5 cells. As shown in Fig. 2*F*, AICAr induced phosphorylation of AMPK at the Thr175 residue 3 and 24 h after the addition of AICAr, and this activation was not observed in cells treated with brequinar.

The antidiabetic biguanide metformin is another widely used AMPK agonist that activates AMPK through a complex pathway involving the mitochondrial phosphorylation (35). Our previous study showed that metformin did not induce differentiation of U937 cells, although it activated AMPK (10). To further test for the role of AMPK in phenotypic changes induced by AICAr in MS-5 cells, the cells were treated with metformin and two specific AMPK activators; 30 µM GSK621 and 10 µM MK-8722. As shown in Fig. 2*G*, all three agents mimic the morphological changes of stromal cells induced by AICAr, particularly 10 µM MK-8722. A decrease in the number of viable cells treated with AICAr, metformin, and MK-8722 was confirmed by counting the number of viable cells after trypsinization (Fig. 2*H*). To further characterize the phenotypic changes induced by AICAr after 3 days of incubation, the expression of conventional mesenchymal stem/stromal cell markers Ly6A/E (Sca-1), CD105, and CD140a (PDGFR-α, platelet-derived growth factor receptor alpha) was analyzed by flow cytometry. As shown in Fig. 2*I*, AICAr, GSK621, and MK-8722 significantly decreased the expression of Ly6A/E and CD105, whereas AICAr and MK-8722 increased the expression of CD140a.

### Human Stromal Cell Lines HS-5 and HS-27 Enhance AICAr- and Brequinar-Induced Differentiation of Monocytic Cell Lines, With No Significant Effects of These Inhibitors on the Phenotype of the Stromal Cell Lines

**Figure 3.**
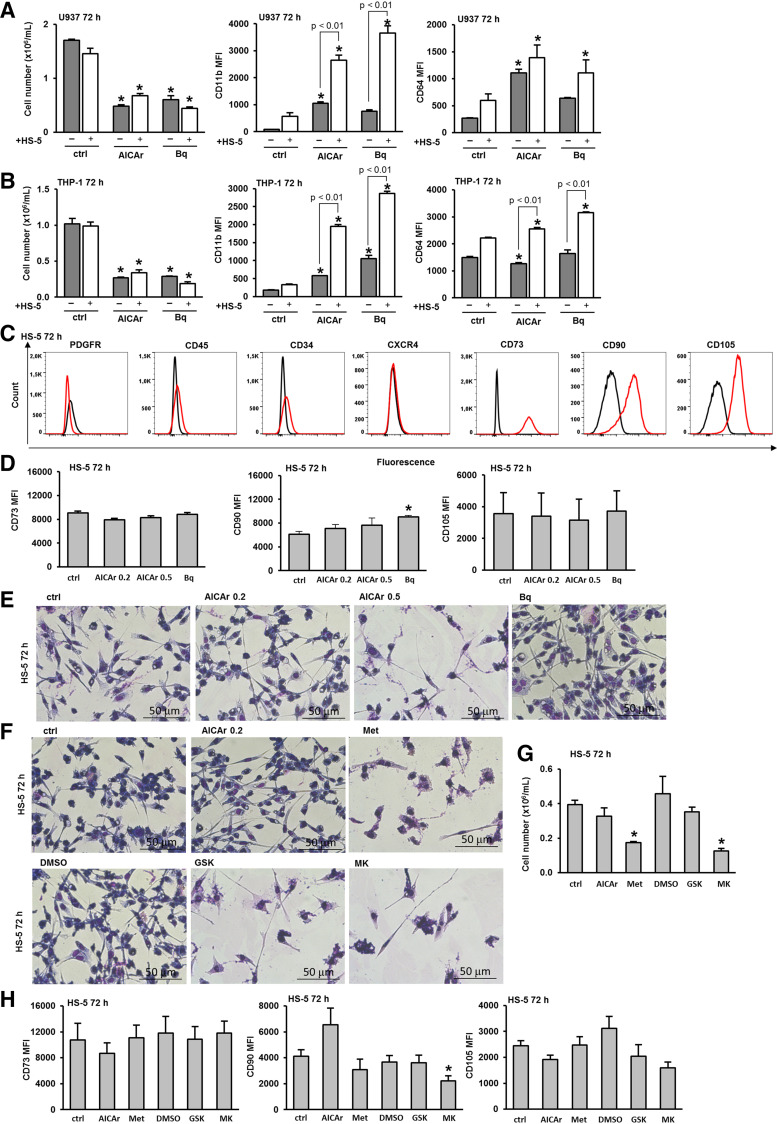
The human stromal cell line HS-5 increases differentiation of U937 and THP-1 cells induced by AICAr and brequinar. U937 and THP-1 cells were cultured either alone or in the presence of the HS-5 stromal cell line and were left untreated (ctrl) or treated with AICAr (0.2 mM) or brequinar (Bq) (0.5 μM) for 72 h. *A*: the number of viable U937 cells and the expression of differentiation markers. *B:* the number of viable THP-1 cells and the expression of differentiation markers. The mean fluorescence intensity (MFI) of CD11b and CD64 was calculated as described in “MATERIALS AND METHODS”. Results represent the means ± SE (error bars) of at least three independent experiments. *P < 0.05 (ANOVA and Tukey) compared with control (ctrl). HS-5 stromal cells were seeded in 6-well plates and allowed to adhere for 24 h. After 24 h, the media were replaced, and the cells were cultured either alone (ctrl) or treated with AICAr (0.2 and 0.5 mM) and brequinar (Bq) (0.5 μM). *C*: immunophenotype of control HS-5 cells after 72 h. Representative histograms are shown, with a red line representing the expression of analyzed marker and a black line representing isotypic control. *D*: immunophenotype of HS-5 cells. *E*: representative images of May-Grünwald-Giemsa stained HS-5 cells (×40 magnification) 72 h after the addition of the tested agents. *F*: representative images of May-Grünwald-Giemsa stained HS-5 cells (×40 magnification) incubated for 72 h with media alone (ctrl), AICAr (0.2 mM), metformin (Met) (15 mM), DMSO (0,1%), GSK-621 (GSK) (30 μM), or MK-8722 (MK) (10 μM). *G*: the number of viable HS-5 cells. *H*: immunophenotype of HS-5 cells analyzed as described in MATERIALS AND METHODS. Results represent the mean ± SE (error bars) of at least three independent experiments. *P < 0.05 (Student’s *t* test) compared with control (ctrl). AICAr, 5-aminoimidazole-4-carboxamide ribonucleoside.

Although the murine bone marrow-derived stromal cell line, MS-5, has been widely used to support both AML cell lines and primary AML samples, murine and human stromal cells are expected to show species-specific differences. Therefore, the effects of AICAr and brequinar were further tested in the presence of the human fibroblast-like cell line HS-5, which is known to support the proliferation of hematopoietic progenitor cells when cocultured in serum-deprived media without exogenous factors (24). The coculture of HS-5 and U937 cells increased the number of viable U937 cells treated with AICAr and significantly increased the expression of CD11b in cells treated with both AICAr and brequinar (Fig. 3*A*). Similarly, coculturing HS-5 with THP-1 cells significantly elevated the expression of CD11b and CD64 in cells exposed to both AICAr and brequinar (Fig. 3*B*). These results demonstrate that the presence of the human stromal cell line does not only inhibit differentiation but also enhances the differentiation effects of pyrimidine synthesis inhibitors.

Next, we tested the potential effects of the inhibitors on the stroma itself. As shown in Fig. 3*C*, HS-5 cells exhibit the typical marker expression profile of mesenchymal stem cells, as defined by the International Society for Cellular and Gene Therapy (ISCT) (36), being strongly positive for CD73, CD90, and CD105. The phenotypic analysis of HS-5 cells treated with two doses of AICAr revealed no significant effects on the expression of CD73, CD90, and CD105. Similarly, no significant effects were observed in cells treated with brequinar, except for a small but significant increase in the expression of CD90 (Fig. 3*D*). As shown in Fig. 3*E*, the addition of AICAr and brequinar at doses previously demonstrated to induce differentiation of AML cells had no effects on the morphology of HS-5 cells after 72 h of incubation. Only the addition of a higher dose of AICAr (0.5 mM) reduced the number of viable cells and induced morphological changes, which were less pronounced than those observed in MS-5 cells. To further investigate possible phenotypic changes induced by AICAr on the stroma itself, we treated HS-5 cells with AMPK agonists. As shown in Fig. 3*F*, metformin, 30 µM GSK621, and 10 µM MK-8722 induced morphological changes similar to those observed with a higher dose of AICAr. The number of viable HS-5 cells was significantly decreased upon treatment with metformin and MK-8722 (Fig. 3*G*). Among AMPK agonists, only MK-8722 exerted significant effects on the expression of CD90, whereas no significant changes in the expression of stromal markers were observed in cells treated with other AMPK agonists (Fig. 3*H*).

**Figure 4.**
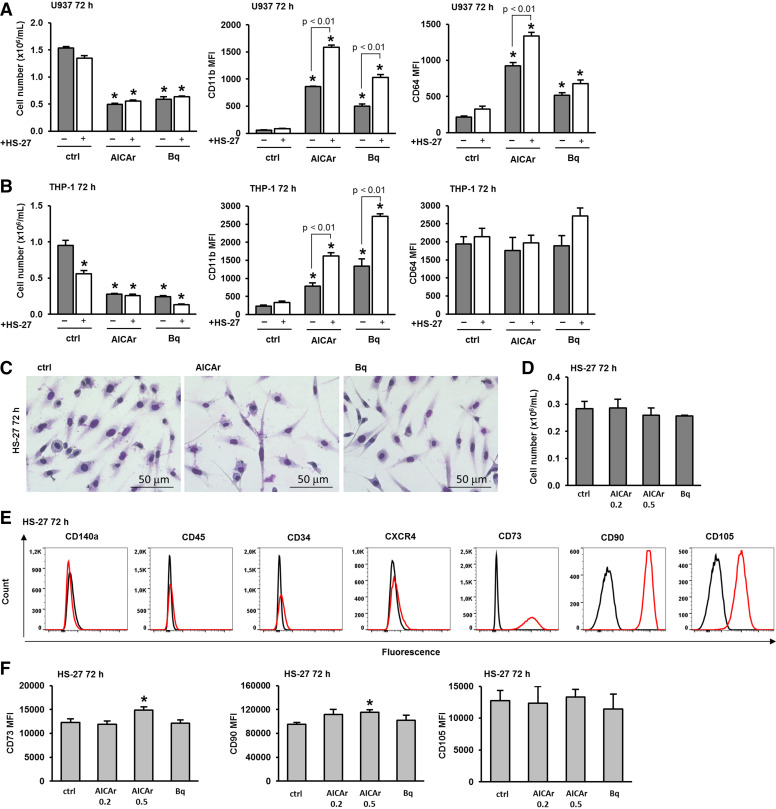
The human stromal cell line HS-27 increases differentiation of U937 and THP-1 cells induced by AICAr and brequinar. U937 and THP-1 cells were cultured either alone or in the presence of the HS-27 stromal cell line and were left untreated (ctrl) or treated with AICAr (0.2 mM) or brequinar (Bq) (0.5 μM) for 72 h. *A*: the number of viable U937 cells and the expression of differentiation markers. *B*: the number of viable THP-1 cells and the expression of differentiation markers. The mean fluorescence intensity (MFI) of CD11b and CD64 was calculated as described in MATERIALS AND METHODS. Results represent the means ± S.E. (error bars) of at least three independent experiments. *P < 0.05 (ANOVA and Tukey) compared with control (ctrl). *C*: representative images of May-Grünwald-Giemsa stained HS-27 cells (×40 magnification) 72 h after the addition of the tested agents. *D*: HS-27 stromal cells were seeded in 6-well plates and allowed to adhere for 24 h. After 24 h, the media were replaced, and the cells were untreated (ctrl) or treated with AICAr (0.2 and 0.5 mM), and brequinar (Bq) (0.5 μM) for 72 h. The number of viable HS-27 cells. *E*: the immunophenotype of control HS-27 stromal cells after 72 h. Representative histograms are shown, with a red line representing the expression of analyzed marker and a black line representing the isotypic control. *F*: immunophenotype of HS-27 cells. Results represent the means ± SE (error bars) of at least three independent experiments. *P < 0.05 (Student’s *t* test) compared with control (ctrl). AICAr, 5-aminoimidazole-4-carboxamide ribonucleoside.

HS-5 is known to support the growth of myeloid colonies and secrete high levels of cytokines that promote myelopoiesis. In contrast, HS-27 is another human stromal cell line with a different cytokine profile that does not support the proliferation of isolated progenitor cells in cocultures (24, 25). To test whether the ability to enhance differentiation in response to pyrimidine synthesis inhibitors is exclusive to the HS-5 cell line, we examined the effects of HS-27 on the differentiation of U937 cells induced by AICAr and brequinar. As shown in Fig. 4*A*, the presence of HS-27 significantly increased the expression of both differentiation markers, CD11b and CD64, in cells treated with AICAr and increased the expression of CD11b in cells treated with brequinar, with no significant effect on the growth arrest induced by these inhibitors. The coculture of HS-27 and THP-1 cells significantly increased the expression of CD11b in cells treated with both AICAr and brequinar (Fig. 4*B*).

Next, we tested the effects of AICAr and brequinar on HS-27 stromal cells themselves. As shown in Fig. 4, *C* and *D*, there were no significant effects on either the morphology or the number of viable HS-27 cells. Similar to HS-5 cells, HS-27 exhibit the typical marker expression profile of mesenchymal stem cells (Fig. 4*E*). Control HS-27 cells displayed a significantly higher expression of all the positive surface markers (CD73, CD90, and CD105) compared with HS-5 cells, as previously described (25). The addition of AICAr and brequinar at doses that efficiently induce differentiation of AML cells had no effect on the expression of stromal cell markers, except that AICAr significantly increased the expression of CD73 and CD90 when applied at a higher dose of 0.5 mM (Fig. 4*F*).

### Primary Human Mesenchymal Stromal Cells Support AICAr- and Brequinar-Induced Differentiation of Monocytic Cell Lines

Although HS-5 and HS-27 are commonly used as reliable models to replicate the biological properties mediated by the mesenchymal stromal cells (MSCs), it is important to note that both cell lines are immortalized cell lines. As such, they may have profoundly altered gene expression profiles and biological properties. To further investigate the effects of human stroma, we isolated MSCs from the bone marrow of patients and a healthy donor and cultured them as previously described (28). MSCs were analyzed at passages 3 or 4 to determine typical immunophenotype according to the minimal criteria for defining MSCs derived from bone marrow (29). These MSCs were then seeded in 12-well plates and cocultured with monocytic cells for 72 h.

**Figure 5.**
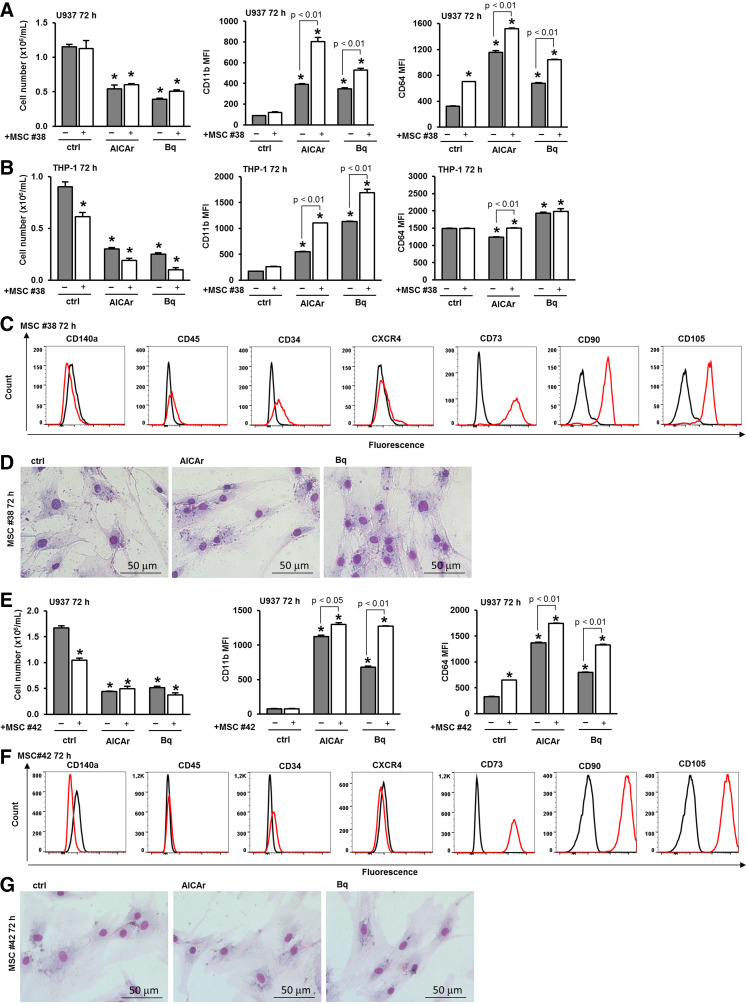
Primary stromal cells from the bone marrow of a non-AML essential thrombocythemia patient (MSC No. 38) and from a healthy donor (MSC No. 42) enhance the differentiation of leukemia cells induced by AICAr and brequinar. U937 and THP-1 cells were cultured either alone or in the presence of stromal cells (MSC No. 38) and were left untreated (ctrl) or treated with AICAr (0.2 mM) or brequinar (Bq) (0.5 μM) for 72 h. *A*: the number of viable U937 cells and the expression of differentiation markers. *B:* the number of viable THP-1 cells and the expression of differentiation markers. The mean fluorescence intensity (MFI) of CD11b and CD64 was calculated as described in “MATERIALS AND METHODS”. Results represent the means ± SE (error bars) of at least three independent experiments. *P < 0.05 (ANOVA and Tukey) compared with control (ctrl). MSCs No. 38 were seeded in 12-well plates and allowed to adhere for 24 h. After 24 h, the media were replaced, and the cells were cultured either alone (ctrl) or treated with AICAr (0.2 mM) and brequinar (Bq) (0.5 μM). *C*: the immunophenotype of control MSCs No. 38 after 72 h. Representative histograms are shown, with a red line representing the expression of analyzed marker and a black line representing the isotypic control. *D*: representative images of May-Grünwald-Giemsa stained MSCs No. 38 (×40 magnification). U937 cells were cultured either alone or in the presence of a healthy donor MSCs (MSC No. 42) and were left untreated (ctrl) or treated with AICAr (0.2 mM) or brequinar (Bq) (0.5 μM) for 72 h. *E*: the number of viable U937 cells and the expression of differentiation markers. The mean fluorescence intensity (MFI) of CD11b and CD64 was calculated as described in MATERIALS AND METHODS. Results represent the means ± SE (error bars) of at least three independent experiments. *P < 0.05 (ANOVA and Tukey) compared with control (ctrl). MSCs No. 42 were seeded in 12-well plates and allowed to adhere for 24 h. After 24 h, the media were replaced, and the cells were cultured either alone (ctrl) or treated with AICAr (0.2 mM) and brequinar (Bq) (0.5 μM). *F*: the immunophenotype of control MSCs No. 42 after 72 h. Representative histograms are shown, with a red line representing the expression of analyzed marker and a black line representing the isotypic control. *G*: representative images of May-Grünwald-Giemsa stained MSCs No. 42 (×40 magnification). AICAr, 5-aminoimidazole-4-carboxamide ribonucleoside; AML, acute myeloid leukemia; MSC, mesenchymal stromal cell.

As illustrated in Fig. 5*A*, the coculture of MSCs isolated from a patient with essential thrombocythemia (ET) (MSC No. 38) and U937 cells increased the number of viable U937 cells treated with brequinar and significantly enhanced the expression of both CD11b and CD64 in AML cells treated with AICAr and brequinar. Similarly, the coculture with MSC No. 38 increased the expression of differentiation markers in THP-1 cells (Fig. 5*B*). As shown in Fig. 5*C*, primary MSCs isolated from a patient with ET expressed the characteristic markers CD73, CD90 and CD105, and lacked the presence of CD45. As shown in Fig. 5*D*, AICAr and brequinar had no significant effects on the morphology and number of MSCs.

MSCs isolated from a healthy donor (MSC No. 42) were also evaluated. As shown in Fig. 5*E*, coculturing with these MSCs increased CD11b and CD64 expression in U937 cells treated with AICAr and brequinar. These healthy donor MSCs similarly expressed the characteristic stromal markers (Fig. 5*F*), and, as shown in Fig. 5*G*, neither AICAr nor brequinar had a significant impact on their morphology.

**Figure 6.**
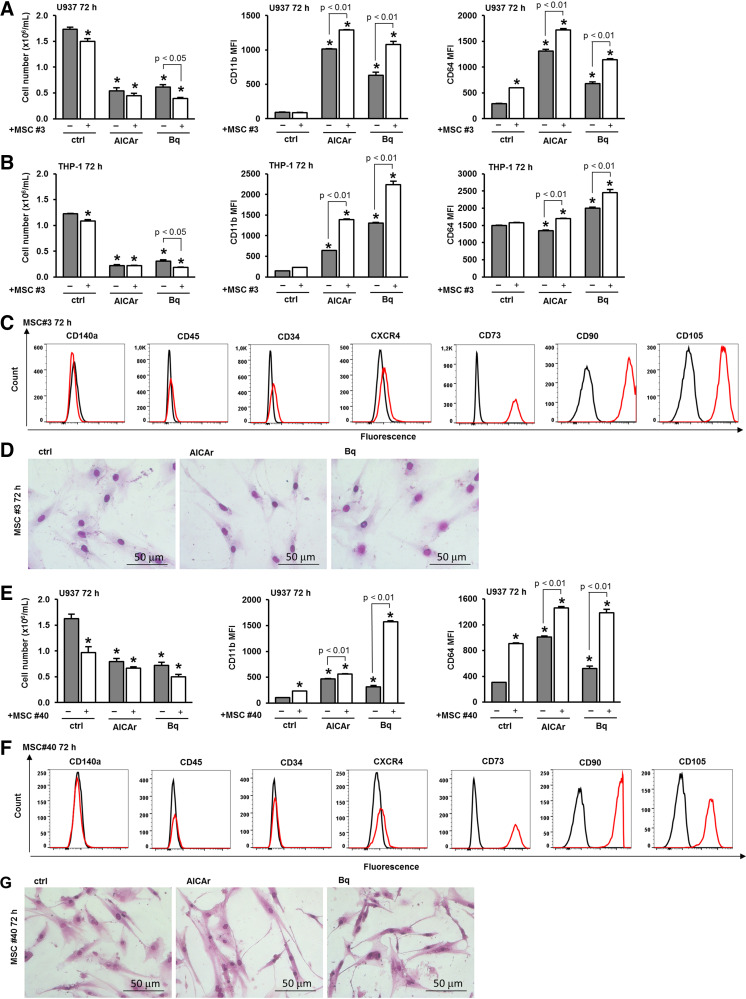
The primary stromal cells from AML-M4 patient BM sample 03 (MSC No. 3) and AML-M0 patient BM sample 40 (MSC No. 40) enhance the differentiation of leukemia cells induced by AICAr and brequinar. U937 cells and THP-1 cells were cultured either alone or in the presence of MSCs No. 3 and were left untreated (ctrl) or treated with AICAr (0.2 mM) or brequinar (Bq) (0.5 μM) for 72 h. *A*: the number of viable U937 cells and the expression of differentiation markers. *B*: the number of viable THP-1 cells and the expression of differentiation markers. The mean fluorescence intensity (MFI) of CD11b and CD64 was calculated as described in MATERIALS AND METHODS. The mean fluorescence intensity (MFI) of CD11b and CD64 was calculated as described in MATERIALS AND METHODS. Results represent the means ± S.E. (error bars) of at least three independent experiments. *P < 0.05 (ANOVA and Tukey) compared with control (ctrl). *C*: the immunophenotype of MSCs No. 3 that were seeded in 12-well plates and allowed to adhere for 24 h. After 24 h, the media were replaced, and the cells were cultured alone. Representative histograms are shown, with a red line representing the expression of CD11b and a black line representing the isotypic control. *D*: representative images of May-Grünwald-Giemsa stained MSCs No. 3 (×40 magnification). *E*: U937 cells were cultured either alone or in the presence of MSCs No. 40 and were left untreated (ctrl) or treated with AICAr (0.2 mM) or brequinar (Bq) (0.5 μM) for 72 h. The number of viable U937 cells and the expression of differentiation markers. The mean fluorescence intensity (MFI) of CD11b and CD64 was calculated as described in MATERIALS AND METHODS. Results represent the means ± SE (error bars) of at least three independent experiments. *P < 0.05 (ANOVA and Tukey) compared with control (ctrl). *F*: the immunophenotype of MSCs No. 40 that were seeded in 12-well plates and allowed to adhere for 24 h. After 24 h, the media were replaced, and the cells were cultured alone. Representative histograms are shown, with a red line representing the expression of CD11b and a black line representing the isotypic control. *G*: representative images of May-Grünwald-Giemsa stained MSCs No. 40 (×40 magnification). AICAr, 5-aminoimidazole-4-carboxamide ribonucleoside; AML, acute myeloid leukemia; BM, bone marrow; MSC, mesenchymal stromal cell.

To further examine the effects of MSCs, we isolated MSCs from two different patients diagnosed with AML. MSCs No. 3 were obtained from the bone marrow of a patient suffering from AML-M4 according to the FAB classification. As shown in Fig. 6*A*, the coculture of MSCs No. 3 and U937 cells increased the expression of differentiation markers in U937 cells induced by AICAr and brequinar. Similarly, coculturing MSCs No. 3 with THP-1 cells enhanced CD11b and CD64 expression in THP-1 cells under the same conditions (Fig. 6*B*). Primary MSCs isolated from a patient with AML-M4 expressed the characteristic stromal cell markers, as shown in Fig. 6*C*, and morphological analysis revealed no changes upon treatment with AICAr and brequinar (Fig. 6*D*).

MSCs No. 40 were isolated from the bone marrow of a patient diagnosed with AML-M0. Once again, the presence of MSCs No. 40 increased the expression of differentiation markers induced by AICAr and brequinar, as shown in Fig. 6*E.* In addition, the immunophenotype of MSCs No. 40 closely resembles the phenotype of both stromal cell lines and other primary MSCs (Fig. 6*F*). Morphological analysis of MSCs isolated from Patient 40 revealed no changes after 72 h of incubation with AICAr and brequinar (Fig. 6*G*).

## DISCUSSION

Several studies have suggested that coculturing AML with bone marrow stromal cells can enhance the biological relevance of drug testing assays compared with monoculture studies. However, both small-scale experiments testing the effects of individual compounds in coculture studies (18, 37, 38) as well as a high-throughput compound screening (17, 39) used assays that measured the effects of drugs on cell viability, apoptosis, and growth arrest, without exploring potential differentiation effects. Tracking differentiation as a novel measure of drug efficacy necessitates methodologies such as multiparameter flow cytometry and morphology assays (16). In our previous study, we found that murine MS-5 cells inhibited differentiation induced by LDAC (22). In the present study, we tested the effects of MS-5 cell line on AML cell differentiation induced by inhibitors of pyrimidine synthesis. We compared MS-5 cells with two human stromal cell lines and primary MSCs isolated from the bone marrow of three different patients. Our findings indicate that the presence of MS-5 cells does not impede differentiation triggered by pyrimidine synthesis inhibitors to the same extent as it inhibits differentiation induced by LDAC. Moreover, the impact of AICAr on CD11b marker expression is slightly enhanced in coculture with MS-5 cells. However, the effect observed with all investigated human stromal cell lines and primary MSCs isolated from patients’ bone marrow demonstrates an even more pronounced increase in the expression of differentiation markers. This suggests that the effects of human stromal cells may differ somewhat from those of mice and likely provide a superior model for examining the effects of stroma on differentiating agents.

The results of our study have confirmed that AICAr has AMPK-dependent and independent effects, and in this coculture model, we have shown that the same concentrations can have different effects depending on the cell type. In our previous work with leukemia cell lines, we established that AICAr facilitates differentiation by inhibiting de novo pyrimidine synthesis and activating the DNA damage signaling pathway, which involves Chk1 kinase (11). Another distinguishing feature of AICAr-induced differentiation in leukemia cells was its biphasic impact on UMP synthesis inhibition and marker expression, with a concentration of 0.2 mM being more effective than 0.5 mM, a phenomenon previously described and attributed to different effects on purine and pyrimidine synthesis (40–42). Conversely, in MS-5 stromal cell lines, such a biphasic effect was absent, as changes in the number and morphology of stromal cells were dose-dependent. Evidently, the effects on the stroma are not mediated by the inhibition of pyrimidine synthesis because, unlike the effects on leukemia cells, they cannot be abolished by the addition of nucleosides; instead, they were mimicked by AMPK agonists.

Morphological changes and significant effects were observed in human stromal cells only in the presence of higher doses of AICAr. The reason why human stromal cells exhibit lower sensitivity to AICAr remains unclear, but it is perhaps advantageous that lower doses of AICAr more effectively promote the differentiation of leukemia cells than they induce changes in human stromal cells at the same time. Although numerous studies have focused on the protective effects of stroma on the cytotoxicity of drugs for leukemia cells, very few studies describe the simultaneous effects on stromal cells. Previous studies have described the effects of high-dose AraC on stroma in vitro (22, 33, 34), and in this work, we confirmed that high doses of cytarabine activate Chk1 in MS-5 stromal cells and reduce the number of viable human stromal cells. However, opinions differ on the role of drug-induced stromal changes in the success of leukemia treatment. In a recent study, concentrations of drugs that impact the stroma were excluded because it was believed that such stromal cells did not provide suitable conditions for coculture (17). However, in other studies, DNA damage to the stroma is considered crucial for establishing a chemoresistant bone marrow niche, as it has been shown that such stroma provides resistance to AML cells against adriamycin and idarubicin-mediated killing through FGF10-FGFR2 signaling (37).

Therefore, AICAr exhibits at least two well-established modes of action; one reliant on the activation of AMPK and the other on the inhibition of UMP synthase. There are numerous reports of both AMPK-dependent and AMPK-independent effects of AICAr across several cell models (5). To our knowledge, the effects of AICAr on bone marrow stromal cells have not been described so far, with only a few reports detailing its effects on fibroblast-like cells. In human hepatic stellate cells and mouse embryonic fibroblasts, it was observed that 1 mM AICAr inhibits proliferation through a mechanism involving mechanistic target of rapamycin (mTOR) inhibition, which is AMPK-dependent, but also causes AMPK-independent S-phase arrest (43). Although the role of nucleosides in this arrest has not been investigated, we can assume that it occurs due to an AMPK-independent effect on the inhibition of pyrimidine synthesis, which is similar to what we observe in leukemia cells differentiating in response to 0.2 mM AICAr. In several models, it has been described that AICAr inhibits the activation of myofibroblasts and the secretion of extracellular matrix, thereby preventing fibrosis, and these effects mostly depend on AMPK (44, 45). The results of our study show that AICAr inhibits proliferation and induces morphological changes in MS-5 cells into fibrocyte-like cells by reducing their size and amount of cytoplasm. These cells resemble metabolically inactive fibroblasts rather than fibrocytes originating from the bone marrow that migrate to injury sites, as they do not express the markers CD34 and CD45, which are typical for bloodborne fibrocytes (46).

The results of our study showed that selective AMPK agonists and metformin affected the number of human stromal cells, but there are no studies showing the effects of AMPK agonists on BM stroma when injected in vivo. In a model of U937 xenografted mice, metformin alone showed no anti-tumor activity, but reduced colonization of AML cells in bone marrow and significantly increased the therapeutic efficacy of AraC in vivo (47). In addition, GSK621 reduced the AML burden in a patient-derived xenograft (PDX) mouse model (7). The morphology of the BM stroma was not reported in these studies. Similar enhancing effects of brequinar on chemotherapy were observed in PDX models in vivo; however, the impact of brequinar on BM stroma was not examined. Coculture studies in vitro suggested that BM stromal cells play a crucial role in providing aspartate, which contributes to pyrimidine synthesis in AML cells, making them more sensitive to brequinar (48). It is possible that the enhancing effect of stromal cells on differentiation induced by pyrimidine synthesis inhibitors in our model is at least partly due to the metabolic microenvironment provided by the stromal cells.

Both AICAr and DHODH inhibitors underwent clinical testing for AML, but there is no data regarding their impact on stroma (49–51). AICAr was initially assessed in B-cell chronic lymphocytic leukemia, exhibiting an acceptable safety profile and demonstrating antileukemic activity among patients with poor prognosis (50). However, in a subsequent trial involving patients with myelodysplastic syndrome and AML, administering AICAr at the same dosage led to a positive response in one patient, alongside notable renal toxicity (51). The maximum tolerated dose of 210 mg/dL used in both studies resulted in a plasma AICAr concentration of 0.9 mM (50). Our research suggests that this concentration is much higher than what is needed for the differentiation of AML cells but is likely sufficient to induce changes in stromal cells in vitro.

In conclusion, the results of our study show that human bone marrow stromal cells enhance differentiation induced by pyrimidine synthesis inhibitors and that AICAr can induce phenotypic changes in mouse stromal cells that do not depend on the inhibition of pyrimidine synthesis. Therefore, although MS-5 cells in coculture are suitable for the study of leukemia-initiating cells and general stem cell biology because they correlate well with the multiple xenotransplantation model in mice (38, 52, 53), they are not ideal for drug testing. As seen from the results of our research, MS-5 is not suitable for essays whose aim is to identify drugs beneficial for a specific strategy, which is the induction of differentiation.

## DATA AVAILABILITY

The authors declare that any supporting data or material associated with this original research is available from corresponding author under reasonable request.

## SUPPLEMENTAL MATERIAL

10.6084/m9.figshare.26976136 Supplemental Table S1: https://doi.org/10.6084/m9.figshare.26976136.

10.6084/m9.figshare.26976139 Supplemental Table S2: https://doi.org/10.6084/m9.figshare.26976139.

10.6084/m9.figshare.26976163 Supplemental Fig. S1: https://doi.org/10.6084/m9.figshare.26976163.

## GRANTS

This work was supported by the Croatian Science Foundation under the project number (HRZZ IP-2022-10–9146), DOK-2018-01-9599, and DOK-2020-01-2873 by the European Union through the ESF Operational Programme Efficient Human Resources (to D.V.), The Lady Tata Memorial Trust, International Award for Research in Leukemia (to V.D.), National Institute of Health Grant R01GM117446 (to A.B.), and Scientific Center of Excellence for Basic, Clinical and Translational Neuroscience, European Regional Development Fund: GA KK01.1.1.01.0007 (to D.V.).

## DISCLOSURES

No conflicts of interest, financial or otherwise, are declared by the authors.

## AUTHOR CONTRIBUTIONS

T.S., H.L., and D.V. conceived and designed research; T.S., H.L., B.T., and D.V. performed experiments; T.S., H.L., V.D., B.T., J.B., R.V., and D.V. analyzed data; T.S., H.L., V.D., J.B., R.V., A.B., and D.V. interpreted results of experiments; T.S., H.L., B.T., and D.V. prepared figures; T.S. and D.V. drafted manuscript; H.L., V.D., B.T., J.B., R.V., and A.B. edited and revised manuscript; T.S., H.L., V.D., B.T., J.B., R.V., A.B., and D.V. approved final version of manuscript.
